# AI-Driven Objective Structured Clinical Examination Generation in Digital Health Education: Comparative Analysis of Three GPT-4o Configurations

**DOI:** 10.2196/82116

**Published:** 2026-01-15

**Authors:** Zineb Zouakia, Emmanuel Logak, Alan Szymczak, Jean-Philippe Jais, Anita Burgun, Rosy Tsopra

**Affiliations:** 1 Clinical Bioinformatics Laboratory Imagine Institute Université Paris Cité, INSERM UMR1163 Paris France; 2 Department of Medical Informatics Hôpital Européen Georges Pompidou, Hôpital Necker Enfants Malades AP-HP Paris France; 3 Imagine Institute Université Paris Cité Paris France; 4 Biostatistic Unit Necker University Hospital AP-HP Paris France; 5 UPPERS US 007 F-75006 Université Paris Cité Paris France

**Keywords:** large language models, generative artificial intelligence, ChatGPT, GPT-4o, medical education, objective structured clinical examination, digital health, digital health education, medical informatics, prompt design

## Abstract

**Background:**

Objective Structured Clinical Examinations (OSCEs) are used as an evaluation method in medical education, but require significant pedagogical expertise and investment, especially in emerging fields like digital health. Large language models (LLMs), such as ChatGPT (OpenAI), have shown potential in automating educational content generation. However, OSCE generation using LLMs remains underexplored.

**Objective:**

This study aims to evaluate 3 GPT-4o configurations for generating OSCE stations in digital health: (1) standard GPT with a simple prompt and OSCE guidelines; (2) personalized GPT with a simple prompt, OSCE guidelines, and a reference book in digital health; and (3) simulated-agents GPT with a structured prompt simulating specialized OSCE agents and the digital health reference book.

**Methods:**

Overall, 24 OSCE stations were generated across 8 digital health topics with each GPT-4o configuration. Format compliance was evaluated by one expert, while educational content was assessed independently by 2 digital health experts, blind to GPT-4o configurations, using a comprehensive assessment grid. Statistical analyses were performed using Kruskal-Wallis tests.

**Results:**

Simulated-agents GPT performed best in format compliance and most content quality criteria, including accuracy (mean 4.47/5, SD 0.28; *P*=.01) and clarity (mean 4.46/5, SD 0.52; *P*=.004). It also had 88% (14/16) for usability without major revisions and first-place preference ranking, outperforming the other configurations. Personalized GPT showed the lowest format compliance, while standard GPT scored lowest for clarity and educational value.

**Conclusions:**

Structured prompting strategies, particularly agents’ simulation, enhance the reliability and usability of LLM-generated OSCE content. These results support the use of artificial intelligence in medical education, while confirming the need for expert validation.

## Introduction

Since ChatGPT was launched by OpenAI in 2022, large language models (LLMs) have undergone rapid development, leading to growing interest in medical education [1-6]. Their ability to produce diverse and context-specific texts from simple prompts offers new opportunities to create and improve educational resources.

In medical education, several studies explored the ability of LLMs to answer medical examination questions [7-12], generate multiple-choice questions [13-16], or simulate patients [17-26], with results considered promising by both educators and learners. However, using LLMs to generate clinical scenarios is still new and has only been explored in a few studies, across three situations:

1. Generation of clinical cases or vignettes (ie, scenarios given to students without interaction): LLMs demonstrated their ability to generate cases considered accurate and appropriate in pharmacology [27], but also useful [28] and usable (with moderate or minor edits) in general medicine [29]. However, the authors noticed some issues with realism [28] and precision [28,29].
2. Generation of clinical simulations (ie, scenarios given to students with interaction, such as simulated patients): LLMs demonstrated their ability to generate accurate cases in asthma [30], as well as realistic cases in the nursing field [31]. They also offered potential time savings, reducing development time by 2.6 hours compared to experts [32]. However, limitations remained, such as the omission of critical patient characteristics [31].
3. Generation of Objective Structured Clinical Examination (OSCE) stations: These scenarios, introduced by Harden and Gleeson in 1979 [33], are key assessment tools in medical education. OSCEs consist of simulated clinical situations conducted within a controlled and reproducible environment [34,35], where students interact with a standardized participant (SP), playing the role of a patient or health care professional. Students are observed and evaluated by an examiner using a standardized scoring rubric, based on predefined criteria. Previous studies explored the potential of LLMs to simulate standardized participants [36-38], examiners [39-43], or students [44-46]. Conceptual work has also discussed their potential to enhance OSCE development [47-49]. However, to our knowledge, no study has yet generated OSCE stations using LLMs.

Designing OSCE stations is a complex and resource-intensive process [50]. It requires considerable clinical and educational expertise to produce realistic, standardized scenarios that accurately assess targeted competencies [51]. The need for precision, clarity, and reproducibility, combined with time and human resource constraints in educational teams, makes OSCE development challenging and burdensome [52,53]. In domains like digital health, this challenge is amplified by a shortage of available experts. In this context, LLMs could help educators by assisting them in creating OSCE stations.

To explore this hypothesis, we aimed to design and assess 3 configurations for generating OSCE stations using ChatGPT in the area of digital health, an emerging field in medical education.

## Methods

### Background

The study follows the recommendations of the METRICS (Model, Evaluation, Timing, Range/Randomization, Individual factors, Count, Specificity) checklist, for designing and reporting studies involving generative language models in medical education [54] (Multimedia Appendix 1).

To analyze the ability of ChatGPT to generate OSCE stations in digital health, we tested 3 configurations of the ChatGPT-4o model (Table 1). These differed by prompt type (simple or advanced) and the documents provided to the LLM (reference books for OSCE or digital health or both).

The configurations of the ChatGPT-4o model are listed as follows:

1. Configuration 1: standard GPT, used the free version of ChatGPT-4o with a simple prompt and the reference book for OSCE construction (named “OSCE Vademecum”).
2. Configuration 2: personalized GPT, used the paid version of custom GPT, with a simple prompt, the OSCE Vademecum, and the reference book on digital health from Université Paris Cité (named “UPCité reference book”).
3. Configuration 3: simulated-agents GPT, used the paid version of custom GPT, with a prompt simulating a fictional multiagent system specialized in OSCE construction (aligned with the OSCE Vademecum), and the UPCité reference book.

**Table 1:** Description of the 3 GPT configurations analyzed.

Characteristics	Standard GPT	Personalized GPT	Simulated-agents GPT
Objective	Simulate minimal and spontaneous use of GPT	Simulate personalized use of GPT with a knowledge base specific to the medical field	Simulate advanced use of GPT with specialized assistants and a knowledge base specific to the medical field
Model	GPT-4o	GPT-4o	GPT-4o
Account type	Free	Paid	Paid
Prompt^a^	Simple	Simple	Simulated multiagent system
OSCE^b^ Vademecum^c^	PDF file	PDF file	Instructions embedded in the prompt
UPCité reference book^d^ (knowledge base)	—^e^	5 Microsoft Word documents	5 Microsoft Word documents

^a^In French (Multimedia Appendix 2).

^b^OSCE: Objective Structured Clinical Examination.

^c^National reference document for designing OSCEs (67-page PDF).

^d^The UPCité reference book was written by 25 educators from 7 health disciplines at Université Paris Cité. It is the reference book for all health students (medicine, pharmacy, nursing, rehabilitation, midwifery, and dentistry). It is divided into 5 booklets: health data, cybersecurity, communication, digital tools in health care, and telehealth (5 Word documents, a total of 222 pages).

^e^Not applicable.

To allow comparison, we provided the same sets of digital health competencies to all 3 configurations, following the process listed in Textbox 1.

**Digital health competencies for configurations.**

- 8 digital health topics were first selected: 5 were single-theme (“Health data, Communication, Cybersecurity, Digital tools, Telehealth”), and 3 were multiple-theme (“Communication and Digital tools”; “Communication, Digital tools, and Cybersecurity”; and “Telehealth, Health data, and Communication”).
- For each selected topic (eg, cybersecurity), 3 digital health competencies were then provided to each configuration (eg, responding to a cyberattack).
  Each configuration then generated an OSCE station related to these predefined competencies.
- In total, 24 OSCE stations were generated (8 topics × 3 configurations), all on the same day, June 1, 2025, during a single 4-hour session.
- Each generated OSCE was in French and included 4 components:
  - The primary and secondary learning domains,
  - The case vignette for the student,
  - The standardized observation checklist for examiners,
  - The script for standardized patients (SP script).

### Description of the 3 Configurations Used to Generate OSCE Stations 

#### Configuration 1: Standard GPT

This configuration aimed to assess ChatGPT’s ability to generate digital health OSCE stations in a minimal configuration, involving a simple prompt and the OSCE Vademecum. It simulated a minimalist and spontaneous use case, akin to an educator working without technical assistance or a reference book.

The configuration description is as follows: (1) Model type: the free version of ChatGPT 4o [55]. (2) Prompt design: a simple prompt relied on a role-prompting strategy. The model was instructed to act as a “digital health expert and instructional designer” and create an OSCE based on 3 given digital health competencies. It was invited to review the OSCE Vademecum to choose learning domains, and then produced the OSCE’s components. No sequential structure or examples were provided, making this a basic, minimalist prompt (Multimedia Appendix 2). (3) Documents used: only the OSCE Vademecum (in PDF format) was provided and served as the reference framework for structuring the OSCE station. (4) Technical configuration: ChatGPT interface, without customization and advanced features.

#### Configuration 2: Personalized GPT

This configuration aimed to assess whether providing a domain-specific knowledge base (here, the reference book in digital health) could improve the quality of the generated OSCE stations. It simulated the use of a personalized GPT by an educator equipped with a reference book.

The configuration description is as follows: (1) Model type: the paid version custom GPT-4o (cGPT) [56], which allows configuring GPT with a specific role using tailored instructions, example queries, and integrated knowledge bases. (2) Prompt design: the simple prompt was similar to Configuration 1, but added an instruction to review the UPCité reference book on digital health (Multimedia Appendix 2). (3) Documents used: both the OSCE Vademecum (in PDF format) and UPCité reference book (in Word format) were provided to cGPT. To optimize readability for the model, the UPCité digital health reference book was preprocessed by removing noneducational pages, simplifying formatting, and converting tables and figures to plain text. (4) Technical configuration:

1. assigning an icon and name to the cGPT
2. inserting instructions in the form of a prompt
3. adding a conversation starter with a user query, here: “I would like to design an OSCE station for assessment in digital health”
4. integrating a knowledge base, here the OSCE Vademecum and UPCité reference book.

None of the optional Custom GPT features (eg, web browsing, code interpreter, data analysis, and image generation) was used.

#### Configuration 3: Simulated-Agents GPT

This configuration aimed to assess whether a rigorous procedural structure, via the simulation of step-by-step simulated agents' reasoning, could improve the quality of the generated OSCE stations. It simulated the use of specialized assistants at each stage of OSCE construction by an educator equipped with a reference book.

Configuration description: (1) Model type: the same as in Configuration 2 (ie, the paid version of cGPT). (2) Prompt design: the prompt was built from instructions extracted from the OSCE Vademecum and structured to simulate a multiagent system. It followed a supervised, sequential, and specialized architecture, functioning like a processing chain in which each agent had a distinct pedagogical role in OSCE generation. The supervisor agent acted as the central coordinator. It collected the 3 competencies provided by the educator, determined the sequence of agent activation, passed contextual information between them, and ensured the overall coherence, without interfering with pedagogical content. After each step, it asked, “Would you like me to proceed to the next agent?” simulating a controlled, step-by-step process.

The following specialized agents were used: (1) The learning domain agent selected a primary and secondary domain from the predefined list included in the OSCE Vademecum, aligned with the learning objectives of the graduate medical curriculum (eg, “Education and Prevention”). (2) The vignette agent generated the case vignette for students, conforming to standardized formatting. (3) The checklist agent created the observation checklist for examiners, following the standardized format. (4) The SP script agent drafted the script for the standardized patient, following the standardized format.

Agents were activated sequentially, each receiving the outputs of the previous steps, and generated a formatted output according to OSCE guidelines and UPCité reference book (Multimedia Appendix 2).

The prompt design combined multiple prompting strategies [48,57]:

1. Role prompting by assigning a specific pedagogical role to each agent;
2. Instruction-based prompting by providing precise, structured instructions drawn from the OSCE Vademecum;
3. Chain prompting by structuring tasks into a sequenced, logical workflow;
4. Few-shot prompting by including illustrations of expected formats to improve consistency and reproducibility.

(3) Documents used: the UPCité reference book on digital health was provided in the cGPT’s knowledge base. (4) Technical configuration: similar setup to Configuration 2 (provision of a name, icon, and prompt, same conversation starter, knowledge base, and no optional features).

### Evaluation of the Quality of OSCE Stations Generated by the 3 Configurations

To compare the 3 configurations, OSCE stations were generated and assessed by experts. The evaluation was conducted blindly of the configuration used, with a comprehensive assessment grid based on a literature review [27-32,58-62].

#### Evaluation of the Format Compliance of the Generated OSCEs

A fifth-year medical student, with a background in computer science and trained in OSCE methodology, measured how well the generated OSCE adhered to the OSCE Vademecum in terms of format compliance.

The compliance checklist included 27 criteria covering (1) the validity of the learning domains, (2) the vignette format, (3) the structure of the observation checklist, and (4) the completeness of the SP script. Each criterion was assessed using a binary scale (Yes or No), focusing only on format (independently of pedagogical quality).

#### Evaluation of the Educational Quality of the Generated OSCEs

A duo of digital health experts, involving the head of the medical informatics ward of “Hôpital Européen Georges Pompidou and Hôpital Necker Enfants Malades AP-HP, and a resident in digital health with a computer science background, conducted the evaluation of the content quality, independently and blindly of the configuration used (each OSCE station was anonymized and randomized).

For each OSCE, 9 evaluation dimensions were assessed:

1. “Relevance of the learning domains,” “Clarity,” “Pedagogical validity,” “Realism,” “Feasibility,” “Educational value,” and “Originality,” which were rated using a 5-point Likert scale of agreement (1: strongly disagree, 2: somewhat disagree, 3: neither agree nor disagree, 4: somewhat agree, and 5: strongly agree).
2. “Information accuracy”, which was rated on a severity scale (1: major issue compromising usability, 2: major issue, 3: moderate issue, 4: minor issue, and 5: no issues identified).
3. “Overall usability,” which was rated on a usability scale (1: not usable, 2: usable with major revisions, 3: usable with minor revisions, and 4: usable as is, without modification).

The criteria used were derived from the literature [27-32,58-61] and from the international OSCE guidelines [62], in order to align with international standards.

At the end of the evaluation process, OSCEs were grouped by similar topic (eg, Cybersecurity). For each group of 3 OSCEs (one per configuration), evaluators were asked to rank them, from first to third, without knowing which configuration had generated each one.

### Statistical Analysis

For format-related criteria, binary variables (Yes or No) were described using frequencies and the percentage of “Yes” responses.

For educational criteria, expert ratings were averaged per OSCE and summarized using means and SD values (n=8 per configuration). Overall usability and ranking were categorical variables, analyzed without averaging expert ratings (n=16 per configuration), and described using frequencies and percentages. Due to the nonparametric distribution of data and the ordinal nature of variables, the Kruskal-Wallis test was used to compare the 3 configurations. For variables comprising multiple sub-criteria, an overall mean score per dimension was calculated and used in the Kruskal-Wallis test to determine statistical significance.

All statistical analyses were performed using the R statistical environment (R version 4.3.3) with RStudio GUI (version 2023.12.1+402; Posit PBC). The threshold for statistical significance was set at *P*<.05. Given the exploratory nature of the study, no correction for multiple testing was performed, with an emphasis on observed trends and effect sizes.

### Ethical Considerations

This study did not involve human subjects. All generated outputs were fictional and intended for educational use. Therefore, in accordance with French Law n°2012-300 [63], no Institutional Review Board approval was required.

## Results

### Format Compliance of the OSCEs Generated

Overall, the simulated-agents GPT showed the highest format compliance, followed by the standard GPT, while the personalized GPT exhibited the lowest performance (Table 2).

Standard GPT OSCEs were fully compliant for the “learning domain” and “vignette” components. The “checklist” showed less compliance (between 3/8, 38% and 4/8, 50%) for the advanced criteria (“distinct items”, “assessment of skill only”, and “clear validation criteria for each item”). The “SP script” component was incomplete for the contextual clinical data of all generated cases, with information, such as symptoms, medications, and socioprofessional background, never present.

Personalized GPT OSCEs were poorly compliant for all components. The compliance was full only for 3 criteria of the “vignette” component (eg, “tasks to perform”).

Simulated-agents GPT OSCEs were fully compliant for the “learning domain,” “vignette,” and “SP script” components. Only 4 items of the “checklist” (“observable items” and the 3 advanced criteria) were partially compliant, ranging from 12% (1/8) to 75% (6/8).

**Table 2:** Evaluation of the format compliance of the Objective Structured Clinical Examination generated by the configuration type.

OSCE^a^ components and criteria		Standard GPT (n=8), n (%)	Personalized GPT (n=8), n (%)	Simulated-agents GPT (n=8), n (%)
**Domain**				
	The selected primary learning domain is included in the list of the 11 domains related to the OSCE assessment	8 (100)	0 (0)	8 (100)
	The selected secondary learning domain is included in the list of the 11 domains related to the OSCE assessment	8 (100)	0 (0)	8 (100)
**Vignette**				
	States the candidate’s role	8 (100)	6 (75)	8 (100)
	Describes the setting	8 (100)	8 (100)	8 (100)
	Introduces the SP’s identity (name, gender, and age)	8 (100)	6 (75)	8 (100)
	Presents the issue or reason for consultation	8 (100)	8 (100)	8 (100)
	Provides tasks to be performed by the student	8 (100)	8 (100)	8 (100)
	Specifies actions the student should not take	8 (100)	0 (0)	8 (100)
**Checklist**				
	Contains 10 to 15 items	8 (100)	2 (25)	8 (100)
	Each item begins with an action verb	8 (100)	5 (62)	8 (100)
	Items are observable	7 (88)	6 (75)	6 (75)
	Items are dichotomous (Yes or No)	8 (100)	5 (62)	8 (100)
	Items are distinct (or grouped if necessary, with precise scoring instructions)	3 (38)	1 (12)	1 (12)
	Assesses only skills (not attitudes or communication)	4 (50)	1 (12)	2 (25)
	Provides clear validation criteria for each item	3 (38)	1 (12)	3 (38)
**SP script components included**				
	Scenario summary	4 (50)	4 (50)	8 (100)
	SP’s mindset or behavior	6 (75)	6 (75)	8 (100)
	Additional data	0 (0)	1 (12)	8 (100)
	Opening sentence	2 (25)	0 (0)	8 (100)
	Identity	7 (88)	6 (75)	8 (100)
	Socioprofessional background – Hobbies	0 (0)	2 (25)	8 (100)
	Personal medical history	1 (12)	1 (12)	8 (100)
	Familial medical history	0 (0)	0 (0)	8 (100)
	Current medications	0 (0)	0 (0)	8 (100)
	Symptoms	0 (0)	0 (0)	8 (100)
	Conditional disclosure of information	8 (100)	7 (88)	8 (100)
	Answers to all the items present in the “checklist” are provided	6 (75)	2 (25)	8 (100)

^a^OSCE: Objective Structured Clinical Examination.

### Educational Quality of the OSCEs Generated

#### Learning Domain Relevance

**Figure 1:**
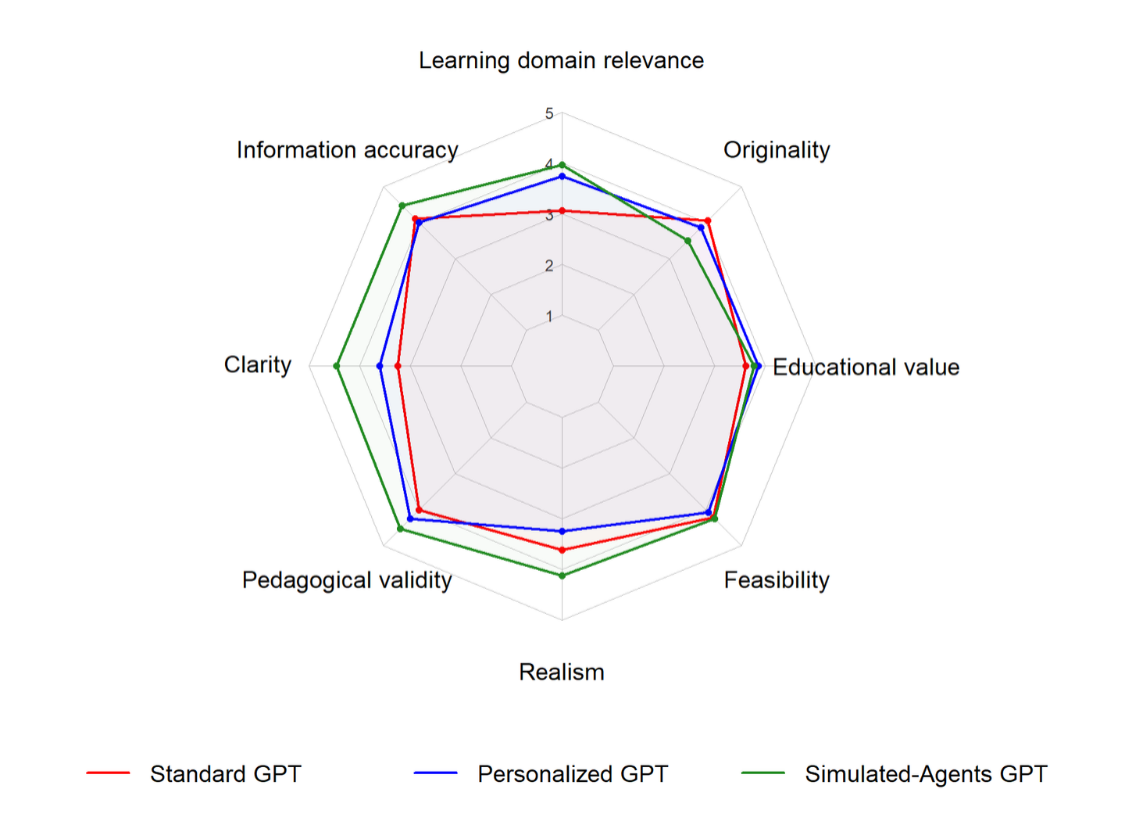
Radar plot of evaluation scores (5-point scales) across the 3 GPT configurations. Agreement-based dimensions (eg, clarity, realism) were rated from 1 (strongly disagree) to 5 (strongly agree); information accuracy used a severity scale from 1 (major issues) to 5 (no issues). The simulated-agents GPT tended to score higher in information accuracy, clarity, realism, pedagogical validity, and learning domain relevance, while it lagged in terms of originality. All 3 configurations showed similar performance for feasibility and educational value.

Primary domain relevance was satisfactory for all configurations, highest for the simulated-agents GPT (mean 4.38, SD 1.38). Secondary domain relevance was more variable, with the standard GPT scoring lowest (mean 2.62, SD 1.36). Differences were not statistically significant (*P*=.16; Tables 3 and 4; Figure 1).

**Table 3:** Evaluation of the content quality of the generated Objective Structured Clinical Examinations, by configuration type.

Evaluation domain, items, and section				Standard GPT (n=8), mean (SD)		Personalized GPT (n=8), mean (SD)		Simulated-agents GPT (n=8), mean (SD)
**Learning domain relevance^a^**								
	Primary domain		3.50 (1.13)		3.94 (1.82)		4.38 (1.38)	
	Secondary domain		2.62 (1.36)		3.56 (1.76)		3.56 (1.18)	
**Information accuracy^b^**								
	**No errors or inexact information**							
		Vignette	3.88 (0.88)		4.38 (0.88)		4.75 (0.46)	
		Checklist	4.06 (0.86)		4.19 (0.75)		4.88 (0.35)	
		SP^c^ script	4.25 (0.85)		3.62 (0.69)		4.19 (0.92)	
	**No missing information**							
		Vignette	4.19 (1.13)		4 (0.60)		4.81 (0.37)	
		Checklist	4.06 (0.82)		3.88 (0.52)		4.62 (0.44)	
		SP script	3.94 (1.02)		3.44 (0.86)		4.19 (0.92)	
	**No irrelevant or unnecessary information**							
		Vignette	4.56 (0.73)		4.62 (0.58)		4.69 (0.37)	
		Checklist	4 (1)		4.31 (0.65)		4.81 (0.26)	
		SP script	4.62 (0.69)		4.38 (0.88)		4.56 (0.73)	
	**No direct cues, leading formulations, or implicit answers**							
		Vignette	3 (1.51)		2.38 (1.75)		2.69 (1.19)	
		SP script	4.56 (0.82)		4.88 (0.35)		5 (0)	
**Clarity^a^**								
	**Clear and comprehensible writing**							
		Vignette	3 (0.96)		3.62 (1.38)		4.44 (0.78)	
		Checklist	3.75 (0.65)		3.62 (0.64)		4.56 (0.56)	
		SP script	3 (0.85)		3.56 (0.98)		4.38 (0.88)	
**Pedagogical validity^a^**								
	**Alignment with the competencies to be assessed**							
		All	3.88 (0.64)		4.06 (0.82)		4.56 (0.42)	
	**Alignment with the expectations of a professional trained in digital health**							
		All	4.12 (0.52)		4.44 (0.68)		4.50 (0.46)	
**Realism^a^**								
	**Realistic situation in a medical professional’s practice**							
		All	3.62 (1.13)		3.25 (0.89)		4.12 (0.69)	
**Feasibility^a^**								
	**Ease of implementation**							
		All	4.19 (0.80)		3.56 (1.35)		4.06 (0.98)	
	**Feasible within an 8-minute timeframe**							
		All	4.19 (0.70)		4.06 (0.78)		4 (0.46)	
	**Ease of recruiting an SP**							
		All	4.25 (0.85)		4.62 (0.88)		4.69 (0.59)	
**Educational value^a^**								
	**Useful for promoting digital health learning**							
		All	3.62 (0.74)		3.94 (0.86)		4.12 (0.88)	
	**Encourages analysis, reflection, and decision-making**							
		All	3.62 (0.88)		3.81 (1.07)		3.44 (0.73)	
**Originality^a^**								
	**Creative potential**							
		All	4.06 (0.50)		3.88 (0.23)		3.50 (0.76)	

^a^Criteria assessed with a Likert scale from 1: strongly disagree to 5: strongly agree.

^b^Criteria assessed with a severity scale from 1: major issues to 5: no issues.

^c^SP: standardized participant.

**Table 4:** Kruskal-Wallis test comparisons of the 3 GPT configurations by evaluation domain. To perform the statistical test, the mean score was calculated for each evaluation domain and configuration. Test results are presented as P values from the Kruskal-Wallis test.

Evaluation domain	Standard GPT, mean (SD)	Personalized GPT, mean (SD)	Simulated-agents GPT, mean (SD)	*P* value^a^
Learning domain relevance	3.06 (1.02)	3.75 (1.75)	3.97 (1.06)	.16
Information accuracy	4.10 (0.36)	4.01 (0.21)	4.47 (0.28)	.01^a^
Clarity	3.25 (0.45)	3.60 (0.83)	4.46 (0.52)	.004^a^
Pedagogical validity	4 (0.5)	4.25 (0.65)	4.53 (0.34)	.12
Realism	3.63 (1.13)	3.25 (0.89)	4.13 (0.70)	.19
Feasibility	4.21 (0.70)	4.08 (0.80)	4.25 (0.51)	.92
Educational value	3.63 (0.65)	3.88 (0.93)	3.78 (0.76)	.75
Originality	4.06 (0.49)	3.88 (0.23)	3.50 (0.76)	.32

^a^*P*<.05 (statistically significant).

#### Information Accuracy

Overall, there was a significant difference in information accuracy (*P*=.01), with the simulated-agents GPT providing the most accurate and comprehensive content (Tables 3 and 4; Figure 1). Specifically,

1. “Errors or inaccurate information” were low across configurations (mean>3.88), with a favorable trend toward the simulated-agents GPT, particularly in the “vignette” (mean 4.75, SD 0.46) and the “checklist” (mean 4.88, SD 0.35) components.
2. “Missing information” was rare in the simulated-agents GPT (mean≥4.19 across all sections), but more common in the personalized GPT, especially in the “script” (mean 3.44, SD 0.86).
3. “Irrelevant or unnecessary information” was rarely noted, with similarly high averages across configurations (mean ≥ 4).
4. “The presence of direct cues or leading formulations” was frequent in the “vignette” (2.38< mean< 3), but nearly absent in the “script”, especially for the simulated-agents GPT (mean 5, SD 0).

#### Clarity

Clarity was significantly different among the 3 configurations (*P*=.004), with a trend in favor of the simulated-agents GPT, getting better results for all components (mean>4.38). The personalized GPT showed intermediate results, while the standard GPT scored lower, particularly for the “vignette” and the “script” (mean 3 for both, SD 0.96 and 0.85, respectively; Tables 3 and 4; Figure 1).

#### Pedagogical Validity

Pedagogical validity was high across all configurations (mean>3.88). Simulated-agents GPT scored highest (mean>4.5), followed by the personalized GPT (mean>4.06), and then the standard GPT (mean>3.88). Although differences were not statistically significant (*P*=.12), a favorable trend for simulated-agents GPT emerged (Tables 3 and 4; Figure 1).

#### Realism and Feasibility

OSCE's realism was satisfactory across configurations, with no statistically significant difference (*P*=.19). The simulated-agents GPT scored the highest (mean 4.12, SD 0.69), followed by the standard GPT (mean 3.62, SD 1.13) and the personalized GPT (mean 3.25, SD 0.89).

No significant difference was observed for the feasibility (*P*=.92). The standard GPT tended to propose OSCEs that were easiest to implement and more feasible to perform within an 8-minute timeframe (mean 4.19, SD 0.70). Ease of recruiting an SP was rated high for all, with a slight advantage for the simulated-agents GPT (mean 4.69, SD 0.59; Tables 3 and 4; Figure 1).

#### Educational Value and Originality

Educational value was comparable across configurations, with no statistically significant differences (*P*=.75). The simulated-agents GPT had the highest score for the use to promote digital health learning (mean 4.12, SD 0.88), while it had a slightly lower score than personalized GPT for encouraging analysis and decision-making (mean 3.44 SD 0.73 vs mean 3.81 SD 1.07 for personalized GPT; Tables 3 and 4; Figure 1).

Regarding originality, there was no significant difference observed (*P*=.32). The standard GPT had the highest average (mean 4.06, SD 0.50), followed by the personalized GPT (mean 3.88, SD 0.23) and the simulated-agents GPT (mean 3.50, SD 0.76; Tables 3 and 4; Figure 1).

#### Overall Assessment

Overall, usability differed significantly across configurations (*P*=.02). For simulated-agents GPT, 88% (14/16) of OSCEs were usable without major revisions, while for standard and personalized GPT, 31% (5/16) of OSCEs required major revisions, and 12% (2/16) were unusable (Table 5).

Overall, generated case rankings differed significantly as well (*P*<.001). The simulated-agents GPT stood out, with 88% (14/16) of OSCEs ranked first and none ranked third. Conversely, the standard GPT ranked third (9/16, 56%) or second (7/16, 44%) but never ranked first. The personalized GPT received a few first-place rankings (2/16, 12%) and was mainly split between second and third places (7/16, 44% each; Table 5).

**Table 5:** Overall assessment of the OSCEs generated by the type of configuration. Test results are presented as P values from the Kruskal-Wallis test.

Assessment domain and items		Standard GPT (n=16), n (%)	Personalized GPT (n=16), n (%)	Simulated-agents GPT (n=16), n (%)	*P* value^a^		
**Usability**						.02^a^	
	Not usable	2 (12)	2 (12)	1 (6)			
	Usable with major revisions	5 (31)	5 (31)	1 (6)			
	Usable with minor revisions	7 (44)	6 (38)	9 (56)			
	Usable as is, without modification	2 (12)	3 (19)	5 (31)			
**Ranking**						<.001^a^	
	First	0 (0)	2 (12)	14 (88)			
	Second	7 (44)	7 (44)	2 (12)			
	Third	9 (56)	7 (44)	0 (0)			

^a^*P*<.05 (statistically significant).

#### Qualitative Feedback

In addition to quantitative ratings, qualitative feedback from experts revealed consistent issues across configurations. All tended to include excessive vignette details, often with leading cues that overly guided students (eg, “Identify and articulate the main risks associated with the use of telemedicine (technical, organizational, legal)”). Inconsistencies were noted between the vignette and SP script, with details missing or contradictory (eg, in the vignette: “Mrs. D […] has also received a message on a secure messaging platform”; in the script: “You do not own a smartphone […] You do not have a computer at home.*”*). Finally, even when highly rated, OSCEs were often considered too theoretical, likely due to reliance on the UPCité digital health booklet, reducing their practical, hands-on applicability for OSCEs.

## Discussion

### Principal Findings

This study compared 3 configurations of GPT for generating OSCE stations in the field of digital health: the standard GPT (simple prompt, OSCE Vademecum); the personalized GPT (simple prompt, OSCE Vademecum, a reference book in digital health), and the simulated-agents GPT (a prompt simulating a fictional multiagent system specialized in OSCEs, a reference book in digital health). Regarding format, the simulated-agents GPT achieved the highest compliance, with minor gaps in advanced checklist criteria. The standard GPT ranked second, with weaker checklist performance and consistently incomplete SP scripts, while the personalized GPT showed the lowest compliance across all components. Regarding educational content, the simulated-agents GPT achieved significantly higher ratings for information accuracy (*P*=.01) and clarity (*P*=.004). It also outperformed in educational validity and realism, although these differences weren’t statistically significant. Additionally, it also had significantly better usability, with 88% (14/16) of the OSCEs usable (*P*=.02). Overall, the simulated-agents GPT significantly outperformed the others, ranking first in 88% (14/16) of the OSCEs and never placing third.

### Interpretation of Results

Differences between the 3 configurations may be explained by several reasons. Regarding format compliance*,* the differences might reflect how each configuration processed and prioritized information. The standard GPT, which used only the OSCE Vademecum, followed the guidelines more closely than the personalized GPT that also included the UPCité reference book. This may be explained by cognitive overload, as adding an extra knowledge source increased retrieval complexity and reduced effective prioritization of key instructions [64]. These issues are amplified by the reliance on a proprietary Retrieval-Augmented Generation (RAG) system that offers only limited transparency and user control [65,66]. The simulated-agents GPT, which used a structured prompt with explicit OSCE guidelines, appeared to improve format adherence. Although it did not fully meet all checklist-format requirements, this suggests that carefully designed prompts are crucial for ensuring format accuracy.

Regarding educational content. The simulated-agents GPT, despite the 8000-character limit, produced high-quality outputs, showing that structured, modular prompts work well even with technical constraints [67]. Interestingly, the standard and personalized GPTs were perceived as more original and more effective at fostering analytical thinking. This suggests a trade-off: while structured approaches improve consistency and adherence to standards, more flexible, broader configurations can boost creativity and originality, albeit with reduced accuracy and lower consistency and control.

Finally, these findings should be interpreted with caution because technology is rapidly evolving [68,69]. The evaluation was based on a dataset generated at a specific time, and since generative models can produce different outputs with the same prompts, reproducibility may be affected. Additionally, the algorithms powering systems like OpenAI's GPT are constantly improving, which could impact future results. However, these considerations do not undermine the validity of our findings. The main goal of the study was to compare 3 OSCE generation strategies, offering insights into how generative models can support medical education rather than advocating for a fixed implementation.

### Limitations

Regarding the LLM used. We focused on ChatGPT, whereas other LLMs have already been used to generate educational resources. For example, Google Bard (now called Gemini Google DeepMind) and Microsoft Bing were used to create multiple-choice questions in medical physiology [70], and LLaMA (Meta AI) was used in radiology [71]. However, prior to designing this study, we conducted preliminary tests with open-source models such as Mixtral-8x7B (Mistral AI) and Llama-3.3-70B combined with custom RAG pipelines. These initial tests were quite disappointing as they had suboptimal performances, particularly for OSCE-style tasks. Notably, recent comparative studies confirm GPT-4's superior performance on RAG-enhanced clinical reasoning tasks [72,73], which reinforces our choice of model despite the broader range of available LLMs.

Regarding the evaluation. It was conducted by 2 experts, which may introduce subjective bias and limit the diversity of evaluative perspectives. However, this limitation is mitigated by their good expertise in digital health and the rigorous evaluation process. A total of 24 OSCEs were reviewed, covering many topics, providing a diverse and valuable dataset. Each OSCE was evaluated with detailed criteria for both format and content, making the results more reliable. A key strength of this study is its strict use of the METRICS checklist [54], a recent guide for studies involving generative models in medical education. This makes the results clearer, easier to reproduce, and comparable. Additionally, while the focus on digital health allowed for targeted exploration in an emerging educational field, it may limit the generalizability of the results to other medical specialties. However, since digital health is growing across many areas, the insight from this work, especially about prompt design and model behavior, is likely useful in other competency-based assessments too.

### Comparison With Other Studies

In the context of OSCEs, other studies used LLMs for tasks other than the generation of OSCE stations:

1. To simulate standardized patients [36-38]. For example, Yamamoto et al [36] used artificial intelligence (AI)–powered chatbots to simulate standardized patients, helping students improve their clinical interview skills. Students working with the chatbots scored significantly higher than those who did not.
2. To automate the OSCE assessment [39-43]. For example, Jamieson et al [41] developed an AI-based grading system to grade post-encounter notes, matching expert scores with 90% agreement while reducing manual grading effort by 91%.
3. To simulate OSCE candidates [44-46]. For instance, Huang et al [44] found that ChatGPT-4.0 performed as well as or better than junior emergency residents in history-taking and record-writing, though humans still demonstrated higher overall consultation quality.

Beyond the context of OSCEs, LLMs have been used to create clinical cases or vignettes for medical education. Like the simulated-agents GPT, studies showed high clarity of the generated content. For example, Coşkun et al [59] reported a 4.11/5 rating for the comprehensibility of the generated scenarios. Additionally, similar to our results, studies also showed strong usability, with Yanagita et al [29] indicating a 97% usability rate for GPT-generated general medicine cases. However, issues with “accuracy” and “missing information” were also reported. For example, Takahashi et al [28] reported 68% accuracy, Yanagita et al [29] reported only 58%, and Scherr et al [30] achieved 100% accuracy, but their evaluation was limited to a single and narrow area (acute asthma) Vaughn et al [31] found that 88% of nursing simulations lacked critical clinical details. In contrast, our simulated-agents GPT consistently yielded complete and context-rich OSCE stations. However, these comparisons should be made carefully, as methodology, design, and metrics differ considerably between studies.

In summary, previous works have largely focused on the use of LLMs to simulate patients, students, or evaluators, or to generate isolated clinical cases. In contrast, to our knowledge, our study is the first to use LLMs to generate OSCE stations, including the vignette, checklist, and SP script.

### Future Directions

This work is part of the DigiHealth Paris Cité project [74]**,** which aims to integrate digital health training into health curricula through innovative methods such as simulation and immersive learning. Here, the objective was to support educators in designing reliable, standardized OSCE stations for student assessment. Based on our findings, the simulated-agents GPT configuration appeared best suited as an assistant for educators, helping to quickly create structured OSCEs that still require validation before introduction into official evaluation sessions.

Regarding technical and pedagogical improvements. The simulated-agents GPT configuration could be further optimized through the refinement of the prompting strategy to enhance output quality and alignment with educational objectives. One effective approach will be iterative development, where expert feedback is continuously integrated into the LLM’s generation cycle. For instance, Coşkun et al [59] used expert prompts to revise and improve 15 clinical cases directly in ChatGPT without manual editing. Similarly, Yanagita et al [29] demonstrated that feeding expert-reviewed vignettes back into the system can help guide and standardize future generations. Therefore, our next steps will include developing an interactive OSCE-generation assistant for educators using the simulated-agents GPT framework, integrating continuous expert feedback to improve content quality, and creating an internal repository of validated AI-generated OSCEs for educators at our faculty, promoting transparency, reproducibility, and shared pedagogical development.

Regarding implementation. The next phase of our work will include a 2-step validation process. First, a pilot testing of expert-validated AI-generated OSCEs with a panel of medical students will assess the realism, usability, and educational value of the generated OSCEs. Second, scaling up to entire student cohorts will enable evaluation of student satisfaction, performance, and learning outcomes at a larger scale. For example, Başaranoğlu et al [58] showed that AI-generated clinical scenario–based questions administered during a urology rotation significantly improved student performance, supporting the educational value of such approaches. Finally, a multisite expansion across other medical faculties is planned to assess transferability. These steps will help translate our findings into practical educational tools that responsibly integrate LLMs into the assessment and training of future medical professionals.

### Conclusions

This study demonstrated the promising potential of LLMs, particularly through a structured, simulated-agents prompting strategy, to create high-quality, usable OSCE stations in the emerging field of digital health. The results highlighted the importance of carefully designed prompts and structured workflows to effectively use LLMs for educational purposes. Finally, our findings suggest that LLMs could be valuable assistants for educators, but expert oversight is still crucial to ensure content quality and relevance of the generated content. Future research should explore the practical application of AI-assisted OSCEs in various educational settings and their integration into medical curricula.

## Appendix

Multimedia Appendix 1 METRICS checklist.

Multimedia Appendix 2 Prompts used to generate the OSCEs for each configuration.

## Abbreviations

AI: artificial intelligence
cGPT: custom GPT-4o
LLM: large language model
METRICS: Model, Evaluation, Timing, Range/Randomization, Individual factors, Count, Specificity
OSCE: Objective Structured Clinical Examination
RAG: retrieval-augmented generation
SP: standardized participant

## Data Availability

The prompts used to generate OSCEs are available in Multimedia Appendix 2. The OSCEs generated during this study are available from the corresponding author on reasonable request.

## References

[1] Preiksaitis C, Rose C (2023). Opportunities, Challenges, and Future Directions of Generative Artificial Intelligence in Medical Education: Scoping Review. JMIR Med Educ.

[2] Aster A, Laupichler MC, Rockwell-Kollmann T, Masala G, Bala E, Raupach T (2025). ChatGPT and other large language models in medical education - scoping literature review. Med Sci Educ.

[3] Gordon M, Daniel M, Ajiboye A, Uraiby H, Xu Ny, Bartlett R, et al (2024). A scoping review of artificial intelligence in medical education: BEME guide No. 84. Med Teach.

[4] Lucas HC, Upperman JS, Robinson JR (2024). A systematic review of large language models and their implications in medical education. Med Educ.

[5] Wu Y, Zheng Y, Feng B, Yang Y, Kang K, Zhao A (2024). Embracing ChatGPT for medical education: exploring its impact on doctors and medical students. JMIR Med Educ.

[6] Zhui L, Yhap N, Liping L, Zhengjie W, Zhonghao X, Xiaoshu Y, et al (2024). Impact of large language models on medical education and teaching adaptations. JMIR Med Inform.

[7] Liu M, Okuhara T, Chang X, Shirabe R, Nishiie Y, Okada H, et al (2024). Performance of ChatGPT across different versions in medical licensing examinations worldwide: systematic review and meta-analysis. J Med Internet Res.

[8] Gilson A, Safranek CW, Huang T, Socrates V, Chi L, Taylor RA, et al (2023). How Does ChatGPT perform on the United States medical licensing examination (USMLE)? The implications of large language models for medical education and knowledge assessment. JMIR Med Educ.

[9] Kung TH, Cheatham M, Medenilla A, Sillos C, De Leon L, Elepaño C, et al (2023). Performance of ChatGPT on USMLE: potential for AI-assisted medical education using large language models. PLOS Digit Health.

[10] Nakao T, Miki S, Nakamura Y, Kikuchi T, Nomura Y, Hanaoka S, et al (2024). Capability of GPT-4V(ision) in the Japanese national medical licensing examination: evaluation study. JMIR Med Educ.

[11] Zong H, Wu R, Cha J, Wang J, Wu E, Li J, et al (2024). Large language models in worldwide medical exams: platform development and comprehensive analysis. J Med Internet Res.

[12] Jaleel A, Aziz U, Farid G, Zahid Bashir M, Mirza TR, Khizar Abbas SM, et al (2025). Evaluating the potential and accuracy of ChatGPT-3.5 and 4.0 in medical licensing and in-training examinations: systematic review and meta-analysis. JMIR Med Educ.

[13] Artsi Y, Sorin V, Konen E, Glicksberg BS, Nadkarni G, Klang E (2024). Large language models for generating medical examinations: systematic review. BMC Med Educ.

[14] Kıyak YS, Emekli E (2024). ChatGPT prompts for generating multiple-choice questions in medical education and evidence on their validity: a literature review. Postgrad Med J.

[15] Cheung BHH, Lau GKK, Wong GTC, Lee EYP, Kulkarni D, Seow CS, et al (2023). ChatGPT versus human in generating medical graduate exam multiple choice questions-a multinational prospective study (Hong Kong S.A.R., Singapore, Ireland, and the United Kingdom). PLoS One.

[16] Abouzeid E, Wassef R, Jawwad A, Harris P (2025). Chatbots' role in generating single best answer questions for undergraduate medical student assessment: comparative analysis. JMIR Med Educ.

[17] Holderried F, Stegemann-Philipps C, Herschbach L, Moldt J, Nevins A, Griewatz J, et al (2024). A generative pretrained transformer (GPT)-powered chatbot as a simulated patient to practice history taking: prospective, mixed methods study. JMIR Med Educ.

[18] Holderried F, Stegemann-Philipps C, Herrmann-Werner A, Festl-Wietek T, Holderried M, Eickhoff C, et al (2024). A language model-powered simulated patient with automated feedback for history taking: prospective study. JMIR Med Educ.

[19] Öncü S, Torun F, Ülkü HH (2025). AI-powered standardised patients: evaluating ChatGPT-4o's impact on clinical case management in intern physicians. BMC Med Educ.

[20] Yu H, Zhou J, Li L, Chen S, Gallifant J, Shi A, et al AIPatient: simulating patients with EHRs and LLM powered agentic workflow. https://arxiv.org/abs/2409.18924.

[21] Wang C, Li S, Lin N, Zhang X, Han Y, Wang X (2025). Application of large language models in medical training evaluation-using ChatGPT as a standardized patient: multimetric assessment. J Med Internet Res.

[22] Stamer T, Steinhäuser J, Flägel K (2023). Artificial intelligence supporting the training of communication skills in the education of health care professions: scoping review. J Med Internet Res.

[23] Li Y, Zeng C, Zhong J, Zhang R, Zhang M, Zou L Leveraging large language model as simulated patients for clinical education. https://arxiv.org/abs/2404.13066.

[24] Liu Y, Shi C, Wu L, Lin X, Chen X, Zhu Y, et al (2025). Development and validation of a large language model-based system for medical history-taking training: prospective multicase study on evaluation stability, human-ai consistency, and transparency. JMIR Med Educ.

[25] Cook DA, Overgaard J, Pankratz VS, Del Fiol G, Aakre CA (2025). Virtual patients using large language models: scalable, contextualized simulation of clinician-patient dialogue with feedback. J Med Internet Res.

[26] Cross J, Kayalackakom T, Robinson RE, Vaughans A, Sebastian R, Hood R, et al (2025). Assessing ChatGPT's capability as a new age standardized patient: qualitative study. JMIR Med Educ.

[27] Sridharan K, Sequeira RP (2024). BMC Med Educ.

[28] Takahashi H, Shikino K, Kondo T, Komori A, Yamada Y, Saita M, et al (2024). Educational utility of clinical vignettes generated in Japanese by chatgpt-4: mixed methods study. JMIR Med Educ.

[29] Yanagita Y, Yokokawa D, Uchida S, Li Y, Uehara T, Ikusaka M (2024). Can AI-generated clinical vignettes in Japanese be used medically and linguistically?. J Gen Intern Med.

[30] Scherr R, Spina A, Dao A, Andalib S, Halaseh FF, Blair S, et al (2025). Novel evaluation metric and quantified performance of ChatGPT-4 patient management simulations for early clinical education: experimental study. JMIR Form Res.

[31] Vaughn J, Ford SH, Scott M, Jones C, Lewinski A (2024). Enhancing healthcare education: leveraging chatgpt for innovative simulation scenarios. Clinical Simulation in Nursing.

[32] Violato E, Corbett C, Rose B, Rauschning B, Witschen B (2023). The effectiveness and efficiency of using ChatGPT for writing health care simulations. ijohs.

[33] Harden RM, Gleeson FA (1979). Assessment of clinical competence using an objective structured clinical examination (OSCE). Med Educ.

[34] Patrício MF, Julião M, Fareleira F, Carneiro AV (2013). Is the OSCE a feasible tool to assess competencies in undergraduate medical education?. Med Teach.

[35] Majumder MA A, Kumar A, Krishnamurthy K, Ojeh N, Adams OP, Sa B (2019). An evaluative study of objective structured clinical examination (OSCE): students and examiners perspectives. Adv Med Educ Pract.

[36] Yamamoto A, Koda M, Ogawa H, Miyoshi T, Maeda Y, Otsuka F, et al (2024). Enhancing medical interview skills through ai-simulated patient interactions: nonrandomized controlled trial. JMIR Med Educ.

[37] Pereira DSM, Falcão F, Nunes A, Santos N, Costa P, Pêgo JM (2023). Designing and building OSCEBot ® for virtual OSCE - performance evaluation. Med Educ Online.

[38] Hirosawa T, Yokose M, Sakamoto T, Harada Y, Tokumasu K, Mizuta K, Shimizu Taro (2025). Utility of Generative Artificial Intelligence for Japanese Medical Interview Training: Randomized Crossover Pilot Study. JMIR Med Educ.

[39] Luordo D, Torres Arrese M, Tristán Calvo C, Shani Shani KD, Rodríguez Cruz LM, García Sánchez FJ, Lagares Gómez-Abascal A, Rubio García R, Delgado Jiménez J, Pérez Carreras M, Diez Lobato R, Granizo Martínez Jj, Tung-Chen Y, Villena Garrido Mv (2025). Application of Artificial Intelligence as an Aid for the Correction of the Objective Structured Clinical Examination (OSCE). Applied Sciences.

[40] Geathers J, Hicke Y, Chan C, Rajashekar N, Sewell J, Cornes S, et al Benchmarking generative AI for scoring medical student interviews in objective structured clinical examinations (OSCEs). https://arxiv.org/abs/2501.13957.

[41] Jamieson AR, Holcomb MJ, Dalton TO, Campbell KK, Vedovato S, Shakur AH, Kang S, Hein D, Lawson J, Danuser G, Scott Dj (2024). Rubrics to Prompts: Assessing Medical Student Post-Encounter Notes with AI. NEJM AI.

[42] Shakur AH, Holcomb MJ, Hein D, Kang S, Dalton TO, Campbell KK, et al Large language models for medical OSCE assessment: a novel approach to transcript analysis. https://arxiv.org/abs/2410.12858.

[43] Tekin M, Yurdal MO, Toraman Ç, Korkmaz G, Uysal İ (2025). Is AI the future of evaluation in medical education?? AI vs. human evaluation in objective structured clinical examination. BMC Med Educ.

[44] Huang T, Hsieh PH, Chang Y (2024). Performance comparison of junior residents and ChatGPT in the objective structured clinical examination (OSCE) for medical history taking and documentation of medical records: development and usability study. JMIR Med Educ.

[45] Li SW, Kemp MW, Logan SJS, Dimri PS, Singh N, Mattar CNZ, et al (2023). ChatGPT outscored human candidates in a virtual objective structured clinical examination in obstetrics and gynecology. Am J Obstet Gynecol.

[46] Aqavil-Jahromi S, Eftekhari M, Akbari H, Aligholi-Zahraie M (2025). Evaluation of correctness and reliability of GPT, bard, and bing chatbots' responses in basic life support scenarios. Sci Rep.

[47] Tsang R (2023). Practical applications of ChatGPT in undergraduate medical education. J Med Educ Curric Dev.

[48] Maaz S, Palaganas JC, Palaganas G, Bajwa M (2024). A guide to prompt design: foundations and applications for healthcare simulationists. Front Med (Lausanne).

[49] Misra SM, Suresh S (2024). Artificial intelligence and objective structured clinical examinations: using ChatGPT to revolutionize clinical skills assessment in medical education. J Med Educ Curric Dev.

[50] Bearman M, Ajjawi R, Bennett S, Boud D (2021). The hidden labours of designing the objective structured clinical examination: a practice theory study. Adv Health Sci Educ Theory Pract.

[51] Khan KZ, Ramachandran S, Gaunt K, Pushkar P (2013). The objective structured clinical examination (OSCE): AMEE Guide No. 81. Part I: an historical and theoretical perspective. Med Teach.

[52] Armijo-Rivera S, Fuenzalida-Muñoz B, Vicencio-Clarke S, Elbers-Arce A, Bozzo-Navarrete S, Kunakov N, et al (2025). Advancing the assessment of clinical competence in Latin America: a scoping review of OSCE implementation and challenges in resource-limited settings. BMC Med Educ.

[53] Ataro G, Worku S, Asaminew T (2020). Experience and challenges of objective structured clinical examination (OSCE): perspective of students and examiners in a clinical department of ethiopian university. Ethiop J Health Sci.

[54] Sallam M, Barakat M, Sallam M (2024). A preliminary checklist (METRICS) to standardize the design and reporting of studies on generative artificial intelligence-based models in health care education and practice: development study involving a literature review. Interact J Med Res.

[55] (2025). ChatGPT. OpenAI.

[56] (2025). ChatGPT: explore GPTs. OpenAI.

[57] Meskó B (2023). Prompt engineering as an important emerging skill for medical professionals: tutorial. J Med Internet Res.

[58] Başaranoğlu M, Akbay E, Erdem E (2025). AI-generated questions for urological competency assessment: a prospective educational study. BMC Med Educ.

[59] Coşkun Ö, Kıyak YS, Budakoğlu Iİ (2025). ChatGPT to generate clinical vignettes for teaching and multiple-choice questions for assessment: a randomized controlled experiment. Med Teach.

[60] Ahmed A, Kerr E, O'Malley A (2025). Quality assurance and validity of AI-generated single best answer questions. BMC Med Educ.

[61] Arain SA, Akhund SA, Barakzai MA, Meo SA (2025). Transforming medical education: leveraging large language models to enhance PBL-a proof-of-concept study. Adv Physiol Educ.

[62] Khan KZ, Gaunt K, Ramachandran S, Pushkar P (2013). Med Teach.

[63] (2025). Titre II : Recherches impliquant la personne humaine (Articles L1121-1 à L1128-12) [Webpage in French]. Légifrance.

[64] Upadhayay B, Behzadan V, Karbasi A Cognitive overload attack:prompt injection for long context. https://arxiv.org/abs/2410.11272.

[65] Knowledge in GPTs. OpenAI Help Center.

[66] (2025). Retrieval augmented generation (RAG) and semantic search for GPTs. OpenAI Help Center.

[67] Key guidelines for writing instructions for custom GPTs. OpenAI Help Center.

[68] Clusmann J, Kolbinger FR, Muti HS, Carrero ZI, Eckardt J, Laleh NG, et al (2023). The future landscape of large language models in medicine. Commun Med (Lond).

[69] Wang Z, Chu Z, Doan TV, Ni S, Yang M, Zhang W (2024). History, development, and principles of large language models: an introductory survey. AI Ethics.

[70] Agarwal M, Sharma P, Goswami A (2023). Analysing the applicability of ChatGPT, bard, and bing to generate reasoning-based multiple-choice questions in medical physiology. Cureus.

[71] Mistry NP, Saeed H, Rafique S, Le T, Obaid H, Adams SJ (2024). Large language models as tools to generate radiology board-style multiple-choice questions. Acad Radiol.

[72] Vaid A, Lampert J, Lee J, Sawant A, Apakama D, Sakhuja A, et al Natural language programming in medicine: administering evidence based clinical workflows with autonomous agents powered by generative large language models. https://arxiv.org/abs/2401.02851.

[73] Ke YH, Jin L, Elangovan K, Abdullah HR, Liu N, Sia ATH, et al (2025). Retrieval augmented generation for 10 large language models and its generalizability in assessing medical fitness. NPJ Digit Med.

[74] (2025). DigiHealth Paris Cité : la révolution de l’enseignement de la santé numérique [webpage in French]. Faculté de Santé, Université Paris Cité.

