# Supplementary material for: AI-Driven Objective Structured Clinical Examination Generation in Digital Health Education: Comparative Analysis of Three GPT-4o Configurations
Source: JMIR Med Educ. 2026 Jan 15;12:e82116. doi: 10.2196/82116 (PMC12856406; doi:10.2196/82116)
Supplement: Multimedia Appendix 1 [file mededu_v12i1e82116_app1.docx]

**METRICS Checklist**

| **METRICS Item** | **Description** | **Response** |
| --- | --- | --- |
| **#1 Model** | What is the model of the generative AI tool used for generating content, and what are the exact settings for each tool? | ChatGPT 4o was used. The configuration details are provided in methods section *Description of the three configurations used to generate OSCE stations* |
| **#2 Evaluation** | What is the exact approach used to evaluate the content generated by the generative AI-based model and is it an objective or subjective evaluation? | Evaluation was performed by 3 experts using a predefined grid, combining objective format criteria and subjective content quality ratings. |
| **#3a Timing** | When is the generative AI model tested exactly and what are the duration and timing of testing? | All OSCEs were generated on the same day, June 1, 2025, during a single 4-hour session. |
| **#3b Transparency** | How transparent are the data sources used to generate queries for the generative AI-based model? | All prompts and content inputs were transparently based on the OSCE Vademecum and the UPCité digital health reference book |
| **#4a Range** | What is the range of topics tested and are they intersubject or intrasubject with variability in different subjects? | The OSCEs covered 8 different topics in digital health, including 5 single-theme and 3 multi-theme topics, ensuring subject variety. |
| **#4b Randomization** | Was the process of selecting the topics to be tested on the generative AI-based model randomized? | Topics to be tested were preselected for representativeness but not randomized. Nonetheless, they were presented to the experts in a randomized manner. |
| **#5 Individual** | Is there any individual subjective involvement in generative AI content evaluation? If so, did the authors describe the details in full? | The experts independently reviewed the OSCEs and in a blinded manner (no participation or knowledge of the study protocol). Each station was anonymized and randomized. |
| **#6 Count** | What is the count of queries executed (sample size)? | A total of 24 OSCEs were generated (8 per configuration). Each included a vignette, checklist, and standardized participant script. |
| **#7 Specificity of the prompt or language** | How specific are the exact prompts used? Were those exact prompts provided fully? Did the authors consider the feedback and learning loops? How specific are the language and cultural issues considered in the generative AI model? | The prompts are clearly defined and fully provided in Multimedia Appendix 2, tailored to each configuration. They were refined iteratively during pilot testing but no real-time feedback loops were applied during generation. All content was produced in French to align with local medical education practices, ensuring language and cultural relevance. |
